# On Syphilis

**Published:** 1872-04-01

**Authors:** Carl Proegler

**Affiliations:** Aurora, Ill.


### ON SYPHILIS.

BY DR. CARL PROEGLER, AURORA, ILL.

I read with very much interest the article on the unity or duality of the syphilitic virus, by Prof. Andrews; and I may be permitted to contribute my mite in support of the dualistic doctrine.

The older authors on syphilis were obliged to consent to a *possibility of direct infection by way of the lips;* and even Torrella (*et hoc accidit propter mammas infectas, aut faciem, aut os nutricis, seu cujus alterius*). Ternellius (*suvenem mulceiem gallico morbo depravatam, ore exosculare assuetum nullo pr. jovem exercitato costu in morbum gallicum cucidisse*).

After Ricord was obliged to change his view, and by exact experimental demonstration the fact was established that syphilis could be inoculated from every syphilitic virus or secretion of the hard chancre of the broad condylomes as well as of the tonsils, I do believe that very few syphilographs dare to doubt the possibility of an infection pr. orum.

Generally, the original syphilitic ulcer on the lips has such a singular character that it cannot be confounded with an abscess produced by a condylomatous destructive process.

*The latter may have entered ever so largely into ulcerative metamorphosis; it will not enter the tissues so deeply, nor have such a hard, sharp-edged basis surrounding it as the ulcus durum of the lips.*

Great caution must be used in forming a diagnosis from the remaining criterion. Here, as with the ulcus durum, the principle holds good that never a single symptom, but the combination of all the symptoms, should be considered and have the preponderance.

Of great value for the decision here in this place is the seeming absence of *initial syphilitic affection and the presence of an intact hymen in women.* And as even in proved infection *via* the lips, a syphilitic affection may occur afterwards on the genitals in consequence of a syphilitic diathesis, other diagnostic signs must be considered. These are the peculiar course and development of the lymphatic glandular swellings which show themselves very much *sooner in the region of the throat than in the inguinal regions.* Generally the submental glands swell first, especially often in the vicinity of the *spina mentalis inteina,* between the *insertion* of the *musculi genio hyoideus* and *genio-glossus.* Secondly, those glands inflame which are situated more nearly at the angle of the mouth and lower forward, not always the superficial only partially covered by the *platysma-myoideus,* but also even sometimes in the deeper glands which are scattered in the *trigonum* cervicals, and which receive the vasa effer-emtia of the *glandulæ fasciales profundæ.*

In one case where the ulcer was situated on the upper lip, I found on the corresponding left side of the face some small swollen glands in front the ear near the parotis, which glands Lewin termed *glandulæ zygomaticæ.* Only after these glands are more or less inflamed does swelling of the inguinal glands occur, preceded in some cases by swelling of the cubital glands. But here it must not be forgotten that also a scrofulous disease brings about similar glandular inflammation.

Koebner characterizes (page 62 of his Clinical Reports) such ulcerations on the lips and mucous membrane of the pharynx "as the rapidly developing symptoms of the usual later period of syphilis, which may be named *syphilis gallopanic* (galloping syphilis), causing the physician to fear for the life of his patient."

My experience does not at all verify this assertion, as I will illustrate in the following cases. It may be different when not the lips but the finer mucous membrane of the deeper laying pharynx is infected with the original syphilis, as has happened in some cases where infection resulted from catheterizing the tuba eustachia. In the greater number of cases which I have observed the symptoms were not very severe, nor were they accompanied by fever nor complications fraught with danger. But I must remark that such persons were generally very anæmic in appearance, and the cure in most cases was retarded.

(*To be continued.*)

Wasp-stings.—Dr. Drury commends (*British Med. Journ.*, Sept. 23, 1871) the following treatment in cases of illness produced by the sting of wasps:

A careful examination of the wound should be made with a good pocket lens, and any remnant of the sting removed with a pair of fine-pointed forceps. Laudanum should be applied by means of a cotton-wool swab for at least ten minutes, followed by warm water fomentations. Internally, brandy and hot water should be given at once, and twenty minims of aromatic spirit of ammonia every half hour as long as there is depression. If the mouth or throat be stung, warm flannels should be applied to the neck, and warm inhalations with ether employed. There is sure to be spasm of the rima glottidis in these cases. In no case that I have seen yet would I have given opium internally; I doubt anything but mischief from its use in any of these cases, but I am aware it has been recommended by medical writers. If local pain be not subdued by the application of the laudanum, then I think I would try the effect of hyoscyamus poultice or tincture of belladonna sprinkled over a warm damp flannel, and applied to the wound.

